# An Electrospun Scaffold Loaded with an Enteromorpha Polysaccharide for Accelerated Wound Healing in Diabetic Mice

**DOI:** 10.3390/md20020095

**Published:** 2022-01-24

**Authors:** Lili Guo, Na Guan, Wenjun Miao, Wenwen Zhao, Qiu Li

**Affiliations:** 1 Agricultural Bio-Pharmaceutical Laboratory, Qingdao Agricultural University, Qingdao 266109, China; guolili_house@126.com (L.G.); qingnong619@126.com (N.G.); miaowenjun97@163.com (W.M.); 2 College of Veterinary Medicine, South China Agricultural University, Guangzhou 510642, China; 3 School of Basic Medicine, Qingdao University, 308 Ningxia Road, Qingdao 266021, China; wenwenzhao0313@163.com

**Keywords:** enteromorpha polysaccharide, anti-inflammatory, diabetic wound healing

## Abstract

The design and development of innovative multifunctional wound dressing materials in engineered biomaterials is essential for promoting tissue repair. In this study, nanofibrous wound dressing materials loaded with anti-inflammatory ingredients were manufactured by a promising electrospinning strategy, and their capability for treating diabetic wounds was also investigated. A scaffold blend consisting of an Enteromorpha polysaccharide and polyvinyl alcohol (PVA) was fabricated. The in vitro and in vivo studies confirmed the efficacy of PVA/EPP1 fiber. We found that PVA/EPP1 fiber accelerated the repair of a full-thickness skin wound in diabetic mice. The results suggest that this scaffold could effectively shorten the wound healing time by inhibiting inflammatory activity, which makes it a promising candidate for the treatment of hard-to-heal wounds caused by diabetes.

## 1. Introduction

Nowadays, hard-to-heal wounds are one of the most severe diabetic complications, ultimately resulting in limb loss and disability. A lot of diabetic foot ulcers (DFUs) occur every year, and approximately 10% of these require amputation surgeries of lower limbs [1]. Wound dressings have been extensively employed to treat diabetic wounds in clinics. Importantly, recent advances have improved the healing efficacy of conventionally-utilized wound dressing materials by incorporating some bioactive ingredients into the dressing materials, containing growth factors [2] and exosomes [3], which have been demonstrated to accelerate endothelial proliferation and angiogenesis. Unfortunately, some demerits, such as the instability of additives and the exorbitant price, severely limit their clinical application.

Generally, wound healing is a well-orchestrated process, referring to several defined phases including hemostasis, inflammation, proliferation, and remodeling [4,5]. Unfortunately, the healing process of diabetic wounds is different from the common wounds, as it is usually disrupted and blocked by a prolonged low-grade inflammation, consequentially slowing down the wound healing [6]. Therefore, modulating the inflammatory response is recognized as a critical factor for promoting wound healing in the early inflammatory stage. The ideal scaffolds can not only modulate inflammation but also support cell adhesion, proliferation, and so on. Many studies have tried to discover suitable materials for this goal.

The Enteromorpha is a type of seaweed rich in polysaccharides which is commonly found in the sea [7,8]. The Enteromorpha polysaccharide (EPP) has been demonstrated to have many pharmacological properties including antioxidant, antibacterial activities and potential health benefits [9]. Nevertheless, to establish the ideal scaffold using the polysaccharide alone is insufficient because of its solubility. The Polyvinyl Alcohol (PVA) approved as a biodegradable material by the FDA can mix with the polysaccharide [10] and make the polysaccharide into a suitable scaffold [11,12,13,14,15]. Meanwhile, Electrospinning is a versatile tool which can be applied for preparing the scaffolds [16,17]. It provides a biomimetic cellular matrix that supports cell interaction and growth. Therefore, we chose the PVA mixed with the polysaccharide co-electrospinning for the fiber preparation.

**Figure 1:**
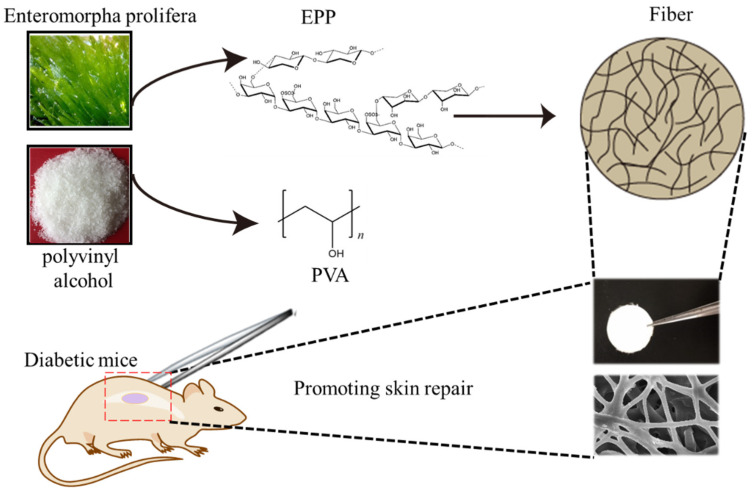
Schematic illustration of the fabricated fiber for skin repair in diabetic mice.

In our study, electrospun nanofiber was chosen as the wound dressing material, which is expected to better mimic the morphology and structure of the existing extracellular matrix (ECM) in the natural skin [18]. We expected that the ECM-mimetic PVA/EPP could inhibit the inflammation in the wound site. With the processing of healing, the fiber can promote tissue repair via enhancing vessel formation (Figure 1). The addition of EPP was expected to construct one multifunctional wound dressing, which could effectively regulate the inflammatory response in the wound sites to enhance diabetic wound healing and skin regeneration.

## 2. Results and Discussion

### 2.1. Physicochemical Characterization of the PVA/EPP Fibers

**Figure 2:**
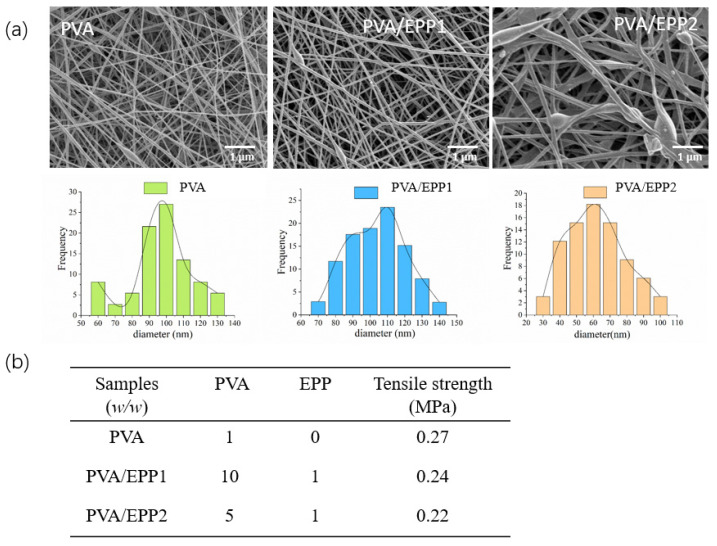
Characterization of nanofibers throughout the sample preparation. (**a**) The morphology of nanofibers loaded with EPP in different concentrations; (**b**) the ratio of EPP.

First, we screened the optimum ratio of PVA to EPP according to the analyzing technology—SEM analysis. We prepared the different fibers containing the different concentrations of EPP polysaccharide. The ratio of EPP and PVA was set from 1:10 to 1:5 (*w*/*w*). The morphology of scaffolds was usually observed by TEM and SEM [19,20]. Then, we selected SEM for the detection of the morphology of PVA/EPP fibers. The results showed PVA/EPP1 fiber was relatively homogeneous and had a diameter ranging from 90 nm to 120 nm. However, with the increasing concentration of EPP, PVA/EPP2 fiber showed discontinuous (Figure 2a). Therefore, we selected PVA/EPP1 fiber for the further study. The mechanical properties of the fibers were characterized by tensile measurement [21]. As for the mechanical property of fibers, according to the result, we can infer that the tensile strength of fibers loaded with EPP is lower than pure PVA fiber. With the increasing content of EPP, the tensile strength of the fibers decreases. The result suggested that the addition of EPP made the fibers more flexible.

Overall, the multifunctional properties of PVA/EPP fibers, including their hollow shape, and their mechanical properties, exhibited the promise of this application in dressing for wound healing.

### 2.2. The Water Absorption of the Fibers

**Figure 3:**
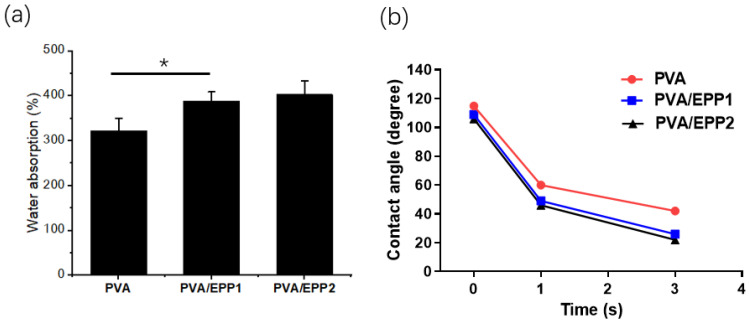
(**a**) Water absorption of nanofiber materials loaded with EPP of different concentrations (* *p* < 0.05; vs. PVA group); (**b**) The water contact angle of PVA/EPP1 at different time points.

We further observed the water absorption properties of PVA/EPP fibers. Meanwhile, the contact angle of the fiber was also measured. From the results, we can conclude that the fiber absorbed water quickly and reached a swelling equilibrium quickly. The fiber of PVA/EPP fibers showed stronger capacity of water absorption compared with pure PVA fiber. The results suggested that the water absorbing capacity increased with the increasing ratio of EPP in the fibers (Figure 3a). Additionally, the contact angle of the fibers was performed at 37 °C. The value of the water contact angle partly depends on the chemical components of the materials [22]. The instant contact angle (ICA) of the fibers sharply decreased from 120 to almost 20 in 3 s (Figure 3b). From the results, we can infer that the addition of EPP makes the fiber more hydrophilic.

In general, according to the obtained results, we can draw a conclusion that PVA/EPP1 fiber has homogenous morphology and a high water absorption capacity for skin repair. These properties are unquestionably beneficial to the wound healing.

### 2.3. Compatibility of the Fiber

**Figure 4:**
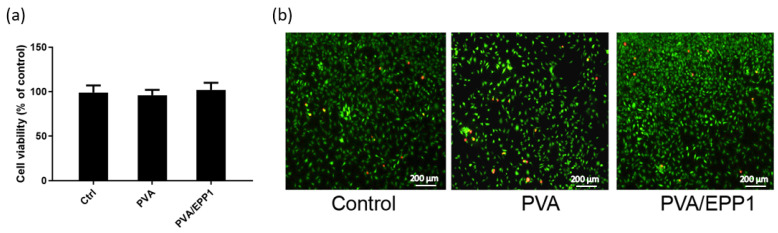
The in vitro evaluation of PVA/EPP1 fiber. (**a**) The effect on cell viability; (**b**) Live/dead staining of L929 cells after treatment with nanofiber membranes for 24 h.

After we accomplished the physical characterizations, PVA/EPP1 was selected for further evaluation in vitro and in vivo. First, we set out to observe compatibility of the fiber on cells. We examined the growth of macrophages on both PVA and PVA/EPP fibers, without or with EPP. The cell compatibility of PVA/EPP1 was evaluated by both MTT assay and live/dead staining (Figure 4a,b). From the results shown in Figure 4, there were no significant differences between all the experimental groups and the control group, and all the fibers have no evident cell cytotoxicity. A similar result was shown in the live/dead staining experiment, which showed that the three groups had nearly the same ratio of live/dead cells (labeled with red fluorescence). Above all, the graphs presented normal morphology (labeled in green fluorescence) in all groups, which demonstrated good compatibility of nanofibers in vitro.

### 2.4. Accerlerated Repair of Wounds by the PVA/EPP1 Fiber

#### 2.4.1. The Fiber Accelerates Cutaneous Wound Healing in Diabetic Mice

**Figure 5:**
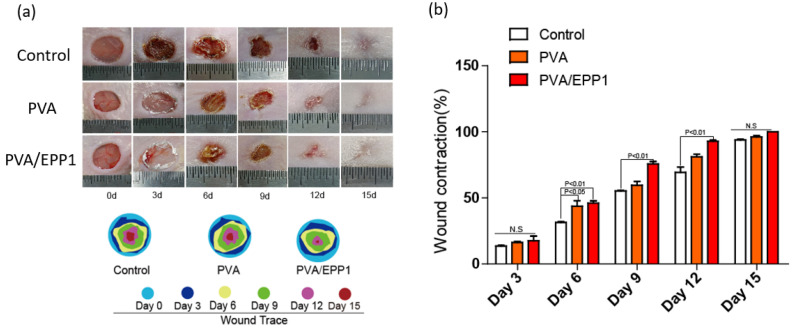
The healing process of diabetic wounds treated by the different fibers. (**a**) Representative graphs of the wound healing process of mice after treatment by different fibers and traces of wound-bed closure at different time points of the different groups; (**b**) Wound closure rates at different time points of the groups treated with the different fibers.

As shown in the above results, PVA/EPP1 fiber possessed multifunctional properties, especially the good compatibility, showed its promise in diabetic wound healing. We examined the wound healing ability of fibers for the full-thickness wounds in mice. The wounds in diabetic mice were covered with the PVA fiber, and PVA/EPP1 fiber, respectively. The untreated wounds in mice were set as the control group. The diabetic wound sizes were photographed at day 0, 3, 6, 9, and 12 after operation. As shown in Figure 5a, the wound surface in all four groups decreased markedly as time increased. Among these, the PVA/EPP1 group exhibited the best healing outcomes, showing complete closure and an inconspicuous scar on day 12, compared with other groups.

Quantitative analysis of the skin repair rate was consistent with the gross morphology. On day 9, the wound contraction rate of the PVA/EPP1 group reached nearly 72%, which was significantly higher than the control group and the PVA group. After 12 days, the wounds closed completely in the PVA/EPP1 group, while wounds still had scarring in other groups (Figure 5a,b).

#### 2.4.2. Histomorphological Analysis and Angiogenesis In Vivo

**Figure 6:**
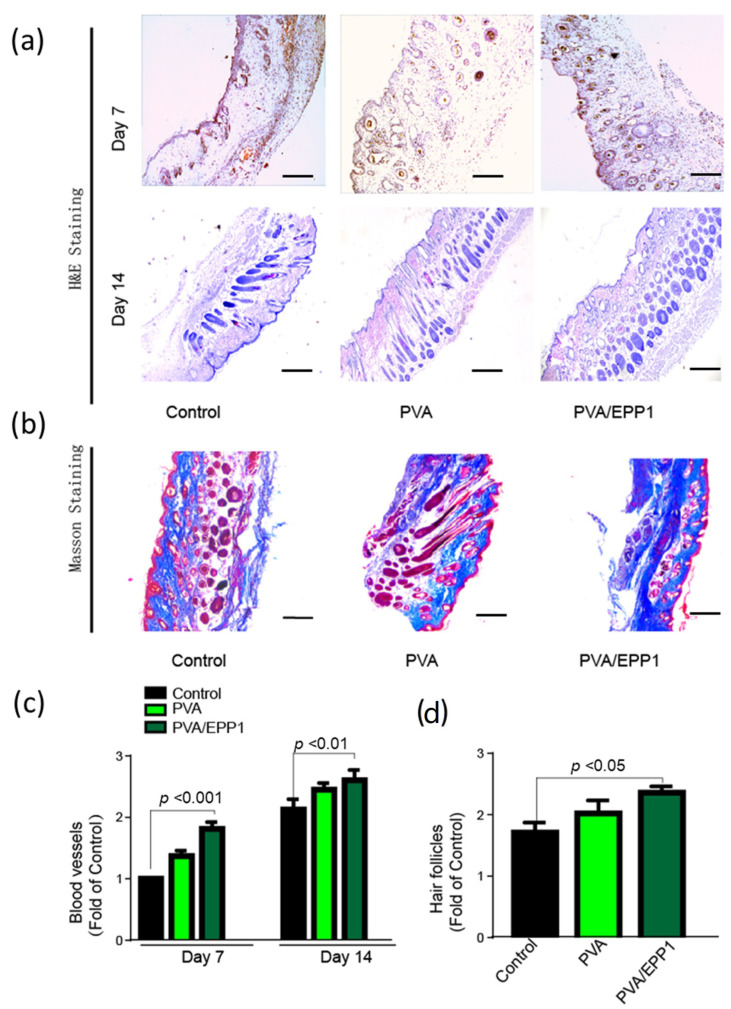
Histological evaluation of diabetic wound healing. (**a**) H&E staining of wounds at day 7 and 14; (**b**) The Masson staining of wounds at day 14, scale bar stands for 500 μm; (**c**,**d**) the number of blood vessels and hair follicles were measured.

We performed the histological analysis with H&E staining. As shown in Figure 6, we can conclude that the epithelization and vascularization recovered better in the PVA/EPP1 group than that in the other groups at day 7 (Figure 6a). After 14 days, the wounds in all the groups were completely closed. Compared with the other two groups, there were more skin hair follicles and blood vessels in the PVA/EPP1 group. In addition, the wound in the PVA/EPP1 group showed more granulation tissue and hair follicles compared with the control group (Figure 6c,d).

During the process of skin repair, collagen deposition plays a vital role in confirming skin scar formation and improving tissue strength in the last stage. Masson trichrome staining was performed in the regenerated skin tissue at day 14. The result was shown in Figure 6b; the relative intensity of collagen stained with blue color in the PVA/EPP1 group was higher than other groups.

### 2.5. The Fiber Modulates the Inflammatory Microenvironment to Promote Angiogenesis

**Figure 7:**
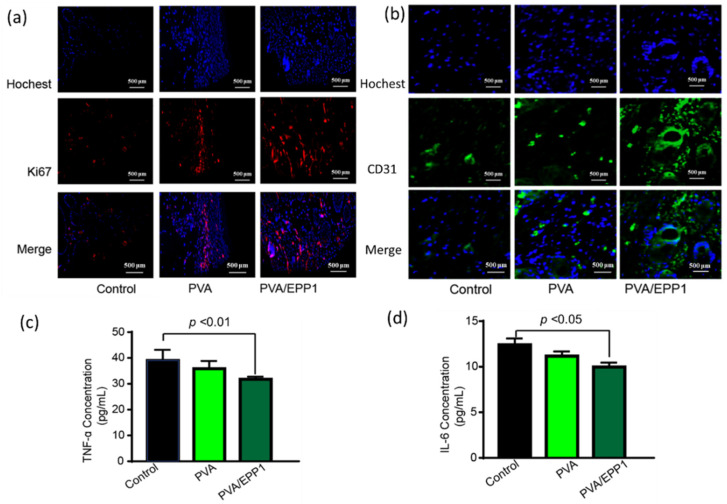
(**a**,**b**) Immunofluorescence staining of CD31 and Ki67 in skin wound tissues at day 14; The concentrations of TNF-α (**c**) and IL-6 (**d**) in serum were measured by ELISA kits.

As we know, the suitable inflammatory microenvironment plays a vital role in wound healing and tissue regeneration. However, the inflammation phase in chronic wounds is severely prolonged, which leads to the delay of the transition into the proliferation phase [23]. In our evaluation of the fibers in vivo, compared with other groups, PVA/EPP1 fiber obviously suppressed the secretion of TNF-α and IL-6, two major inflammatory cytokines, which modulated the wound microenvironment in vivo (Figure 7c,d). This action further induced the transformation of wound from inflammation to proliferation. In order to further explore the regulation of anti-inflammation on tissue regeneration, we observed the formation of new tissues by immunofluorescent staining of the relative markers. We selected two major markers including ki-67 and CD31 at day 14 after implantation. From the results in Figure 7a,b, we can conclude that the tissue in PVA/EPP1 group showed the strongest expression of Ki67. Meanwhile, compared with the other two groups, the expression of CD31 was also strongly strengthened by EPP1 in the PVA/EPP1 group. In sum, all the results suggested that PVA/EPP1 fiber could regulate the inflammatory response in the early stage of wound healing and further promote skin repair.

## 3. Materials and Methods

### 3.1. Materials

Enteromorpha polysaccharide (Purity 95%) was purchased from Qingdao Seawin Biotech Group (Qingdao, China). It was from *Enteromorpha prolifra*, multicellular green algae, and was prepared through digesting, separating, filtrating, concentration, dialysis, sterilization and drying. Poly (vinyl alcohol) with a viscosity of 50–60 mpa.s was obtained from Sinopharm Group (Shanghai, China). All other chemicals and reagents at analytical grade were purchased from Sigma-Aldrich (St. Louis, MO, USA).

### 3.2. Electrospinning Fabrication of PVA/EPP Fibers

PVA/EPP fibers were fabricated by electrospinning technology. Briefly, the PVA was dissolved in 50% acetic acid heating at 85 °C for 3 h and cool to room temperature, then mixed with EPP with vigorous stirring for 15 min. The two solutions were mixed to ensure the ratio of EPP to PVA to be 1:10 and 1:5 (*w*/*w*), named as PVA/EPP1, PVA/EPP1, respectively. Then a 10 mL plastic syringe loaded the obtained solution was used for the electrospinning (TongliWeina, Shenzhen, China). The fibers were collected using an aluminum foil collector at 20 kV. The distance from the syringe to the collector was set at 15 cm and the flow rate was 0.1 mL/h.

### 3.3. Characterization of the Fibers

First, the morphology of the fibers was surveyed using scanning electron microscope (JSM-7500F, JSM, Tokyo, Japan), and the average diameter was calculated using Image J software. The contact angle was observed using contact angle measuring instrument (KRUSS Cluis DSA25, Kruss, Hamburg, Germany). The mechanical tensile tests of fibers were assessed by material testing machine (CTM2500, Xieqiang Co., ltd, Shanghai, China). The water absorption of the scaffold plays a vital role in wound healing [24]. Then, the water absorbing capability of the fiber was measured in PBS (PH = 7.4). Briefly, dry nanofibers were cut into small round pieces and weighed (w0). After that, they were soaked into PBS at 37 °C and weighed (wt) until saturated. Finally, the detailed value was calculated [25].

### 3.4. Cell Growth In Vitro

The cell culture mediums were prepared by immersing the different fibers in a commercialized cell culture medium for 24 h at 37 °C. The mouse fibroblast cell line L929 (ATCC, Manassas, VA, USA) was cultured in Dulbecco’s Modified Eagle Medium (DMEM) with 10% FBS. The cells were seeded onto the fibers and then incubated for 24 h at 37 °C. Cell viability was quantitatively detected by CCK-8 assay. The gross morphology of the cells was determined by calcein-AM staining (10 µM, 15 min) and propidium iodide (5 µM, 15 min) and observed with a confocal microscope (Nikon, Tokyo, Japan).

### 3.5. Establishment of a Diabetic Full-Thickness Wound Model

In this study, animal protocols were approved by the Animal Care and Use Committee of Qingdao University. The animal feeding conformed to the Guidelines for the Care and Use of Laboratory Animals, which published by the National Institutes of Health (NIH Publication No. 8023, revised 1978). The BALB/c mice (8-week-old, 21–26 g) used in this study were kept in a room at 24 °C with a 12-h light and dark cycle. Then, the mice were injected with 1% streptozotocin (STZ, 65 mg/kg) to establish a diabetic model. After five days, the whole-blood glucose obtained from the tail vein was monitored using a glucose meter (Johnson, shanghai, China). After that, the diabetic mice were anesthetized and cut off with one circular full-thickness skin wound (10 mm diameter) under the sterile conditions. Then the wounded mice were randomly divided into three groups, the control group, PVA group and PVA/EPP1 group (mice, *n* = 6). The mice were raised in different cages.

### 3.6. Gross View of Wound Healing

After the operation, we observed the gross morphology of the wounds at the different time points. The rate of wound healing was summarized according to comparison with the original area of wound. For each group, we took a 5 mm surrounding intact tissue around the wound. The skin tissues removed from the wound bed of the mice sacrificed at day 7 and day 14 were fixed in 4% paraformaldehyde for 24 h. The fixed tissues were embedded in paraffin, sectioned at a thickness of 5 μm, and stained with H&E staining and Masson staining for histological analysis using the corresponding kits according to the manufacturer’s instructions (Jiancheng Biotech Co., Nanjing, China).

### 3.7. Immunofluorescence Analysis (IF) Staining of the Tissue Sections

For immunofluorescence analysis, paraffin sections of the wound tissue samples from the control, PVA and PVA/EPP1 groups were first dehydrated, boiled in sodium citrate buffer for about 20 min. After washing in PBST, anti-CD31 and anti-Ki67 (4A Biotechnology Co., Ltd., Beijing, China) were incubated with the tissues for 12 h at 4 °C. The sections were washed three times with PBS and then sealed with an anti-fluorescence quenching tablet containing 4′,6-diamidino-2-phenylindole (DAPI, Solarbio, Beijing, China). The slides were examined with a fluorescence microscope (Nikon A1 MP, Tokyo, Japan). Quantitative analysis was performed using Image J software.

### 3.8. ELISAs for Cytokine Detection

On day 3, the blood samples were collected, which were centrifuged at 1000× *g* for 5 min at 4 °C to obtain the serum. The concentrations of the inflammatory cytokines TNF-α and IL-6 in the serum were determined using the corresponding ELISA kits (4A Biotechnology Co., Ltd., Beijing, China).

### 3.9. Statistics

All data are presented as mean ± standard deviation (SD). Statistical analyses were performed using ANOVA (GraphPad Prism 6), with *, **, and *** standing for *p* < 0.05, *p* < 0.01, and *p* < 0.001 respectively.

## 4. Conclusions

In this study, a multifunctional PVA/EPP1 nanofiber was successfully fabricated and exhibited excellent water absorbing properties and potent anti-inflammatory activity. Compared with the PVA fiber, the PVA/EPP1 fiber shortened the inflammatory period, and enhanced cell proliferation and tissue regeneration. Overall, the PVA/EPP1 fiber shows great potential as a multifunctional wound dressing material for full-thickness diabetic wound repair, which represents a useful strategy in tissue engineering.

## Data Availability

Not applicable.
